# Long‐Term Survival With Olaparib Maintenance Therapy in Metastatic Pancreatic Carcinoma of a Patient Harboring Germline BRIP1 and ATM Mutations

**DOI:** 10.1155/crom/9962240

**Published:** 2025-12-16

**Authors:** Ruemu E. Birhiray, Maya N. Birhiray, Samuel L. Ranger, Vincent L. Flanders

**Affiliations:** ^1^ Indy Hematology Education, Inc., Carmel, Indiana, USA; ^2^ Hematology Oncology of Indiana, Indianapolis, Indiana, USA; ^3^ Vascular and Interventional Physicians, Northwest Radiology, Carmel, Indiana, USA

## Abstract

Pancreatic ductal adenocarcinoma (PDAC) is one of the deadliest cancers in the United States, causing approximately 50,000 deaths annually. Among PDAC patients, those with germline BRCA1/2 mutations show a more favorable response to platinum‐based chemotherapy and PARP inhibitors like olaparib. The 2019 randomized placebo‐controlled double‐blind Phase 3 POLO trial demonstrated olaparib′s efficacy as a first‐line maintenance therapy in patients with BRCA‐mutated metastatic PDAC following platinum‐based chemotherapy. Olaparib was subsequently approved by the FDA, EMA, and PMDA. However, this treatment approach has not been extended to other homologous recombination deficiency (HRD)–related mutations. This case report details a 72‐year‐old white, female patient with cogermline mutations in the ATM and BRIP1 genes, both of which are involved in DNA repair pathways, resulting in HRD. Following a diagnosis of metastatic PDAC, the patient achieved complete remission after retreatment with FOLFIRINOX and has maintained remission for over 40 months on olaparib maintenance therapy. Her ongoing remission, coupled with undetectable levels of circulating tumor DNA, supports olaparib′s potential effectiveness in HRD‐positive PDAC beyond BRCA mutations. This case highlights the need for expanded HRD testing and consideration of PARP inhibitor maintenance therapy for PDAC patients with HRD pathway deficiencies. Our findings advocate for further clinical studies to assess the broader applicability of PARP inhibitors in PDAC patients with HRD mutations, including ATM and BRIP1, which could enhance survival outcomes in this high‐risk population. Expanding the standard of care to include PARP inhibitors for HRD‐positive PDAC could address a critical gap in treatment and improve patient prognosis.

## 1. Introduction

Pancreatic ductal adenocarcinoma (PDAC) is a major cause of cancer‐related deaths in the United States, with around 50,000 annual deaths [1]. However, a small subgroup of PDAC patients with germline *BRCA1/2* mutations have a better prognosis due to their sensitivity to platinum‐based chemotherapy and the availability of PARP inhibitors [2]. In 2019, a PARP inhibitor called olaparib, tested within the POLO trial on this specific subgroup, was found to be effective as a maintenance therapy, demonstrating a median progression‐free survival of 7.4 months compared to 3.8 months for placebo [3]. This led to the FDA′s approval of olaparib in December 2019, and it is indicated for the maintenance treatment of adult patients with deleterious or suspected deleterious germline BRCA‐mutated (gBRCAm) metastatic pancreatic adenocarcinoma whose disease has not progressed on at least 16 weeks of a first‐line platinum‐based chemotherapy regimen [4]. Patient selection for olaparib in this indication is based on an FDA‐approved companion diagnostic test [5]. The BRACAnalysis CDx test (Myriad Genetic Laboratories Inc.) is the validated assay approved for identifying germline mutations in BRCA1 or BRCA2 genes in patients with pancreatic cancer to determine their eligibility for olaparib treatment.

Olaparib is the standard of care for advanced metastatic PDAC with *BRCA1/2* mutations, but it is not yet approved for *BRCA1/2*‐like neoplasms. Guidelines outlined by the National Comprehensive Care Network (NCCN), American Society of Clinical Oncology (ASCO), and European Society for Medical Oncology (ESMO) all support the use of olaparib for treating PDAC [6–8].

A recent analysis of the Know Your Tumor Registry identified mutations in the DNA damage repair (DDR) pathway in 25% of PDAC patients. The affected patients were divided into three groups: *BRCA1/2* and *PALB2* mutations, *ATM/ATR/ATRX* mutations, and other DDR‐related gene mutations. *BRCA1/2* and *PALB2* mutations were found in 5%–6% of patients with PDAC who were unselected for mutational status, whereas higher rates of 5%–19% were found in the high‐risk populations, such as Ashkenazi Jews and those with family histories of pancreatic, ovarian, or breast cancer [9]. Pathogenic DDR variants can lead to homologous recombination deficiency (HRD), similar to *BRCA1/2* mutations, which could also indicate a potential responsiveness to PARP inhibitors [10]. Consequently, this BRCAness or HRD phenotype is recognized even without *BRCA1/2* mutations, potentially extending the relevance of PARP inhibitors to other genetic backgrounds. PARP inhibitors have been shown to be effective in treating a number of cancers, significantly improving the prognosis in patients with ovarian and prostate HRD mutation cancers [10, 11], indicating that they may be effective in treating a host of cancers that meet the HRD phenotype inclusive of PDAC.

*BRIP1*, also known as *BACH1* or *FANCJ*, interacts with *BRCA1* and plays a role in DNA repair and tumor suppression. Mutations in *BRIP1* are linked to breast and ovarian cancers and can influence sensitivity to chemotherapeutic agents like 5‐fluorouracil (5‐FU) and oxaliplatin [12–16]. Clinical experiences with PARP inhibitors in PDAC patients with *BRIP1* mutations are currently limited, although ongoing research suggests potential therapeutic strategies targeting *BRIP1*. Additionally, the *ATM* gene, frequently mutated in PDAC, also sensitizes patients to DNA‐damaging agents such as platinum‐based chemotherapy and PARP inhibitors [17–22]. Despite limited clinical data, ongoing trials are assessing the effectiveness of targeted therapies for *ATM*‐deficient tumors, with much anticipation for the results.

**Figure 1:**
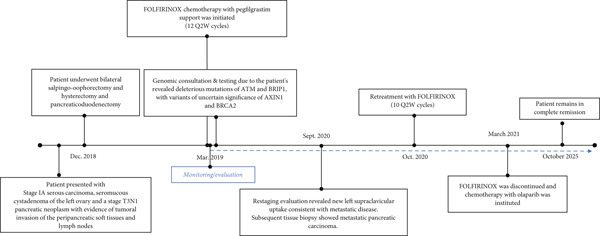
Timeline summarizing the diagnosis and treatment history of a patient with metastatic PDAC harboring BRIP1 and ATM mutations.

In this case report, we detail a case of a patient with cogermline mutations of BRIP1 and the ATM gene who has been successfully maintained on the PARP inhibitor olaparib with ongoing complete remission (Figure 1). The patient described in this case report has been informed that the case will be submitted for publication in *Case Reports in Oncological Medicine* and has provided written informed consent for the publication of identifying patient data, including images and clinical details, in accordance with the journal′s ethical guidelines and applicable privacy regulations. The patient understands that their name will not be disclosed in the publication. All identifying information has been removed or anonymized to protect the patient′s privacy. As a result, this paper is exempt from institutional review board review.

## 2. Results

A 72‐year‐old patient who first presented with bilateral breast carcinoma with negative *BRCA1/2* mutational testing was treated with hormonal therapy until she recurred with an abnormal mammogram 13 years later. She was diagnosed with right‐sided infiltrating ductal carcinoma that was estrogen and progesterone receptor positive and HER‐2/neu negative, which resulted in subsequent bilateral mastectomies and further monitoring. The patient had a family history of Hodgkin′s lymphoma, breast, colon, and lung cancer.

Seven years later, the patient presented with abdominal pain resulting in CT imaging which revealed a large pelvic mass as well as biliary dilatation. Subsequently, the patient underwent bilateral salpingo‐oophorectomy and a Whipple resection which revealed Stage IA serous borderline tumor with rare microinvasion, mucinous cystadenoma of the left ovary and pancreatic adenocarcinoma, pathologic Stage pT3N1, poorly differentiated Grade 3 with negative surgical margins, tumor invasion of the peripancreatic soft tissues, and 14/15 positive lymph nodes. RECIST 1.1 was used to define the initial response, but no evidence of distant metastases was identified as lymph nodes measured less than 10 mm (Figure 2a,d).

Figure 2(a–c) Positron emission tomography (PET) images and (d–f) computed tomography (CT) images. Initial absence of supraclavicular lymphadenopathy (a, d) while the patient was on FOLFIRINOX chemotherapy; 1 year later, the patient develops supraclavicular lymphadenopathy (b, e). Following treatment with FOLFIRINOX chemotherapy and off‐label use of olaparib as maintenance therapy, the patient remains in complete remission after 55 months (c, f). The blue arrows show the presence (b, e) or absence (a, d, c, f) of swollen supraclavicular lymph nodes.

**Figure:**
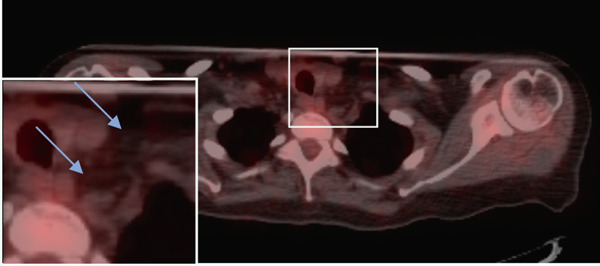
(a)

**Figure:**
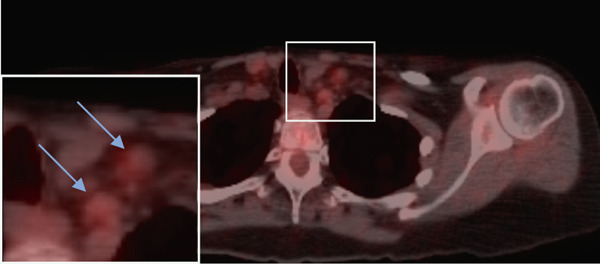
(b)

**Figure:**
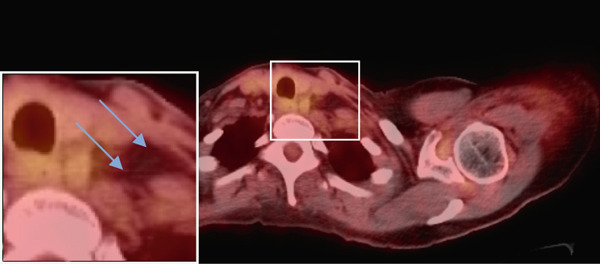
(c)

**Figure:**
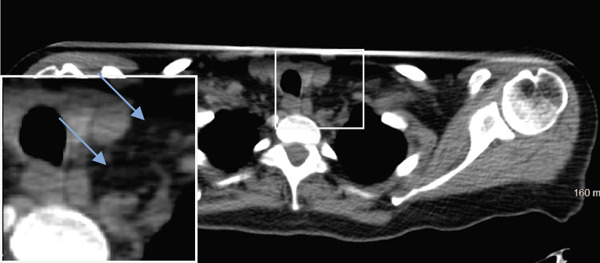
(d)

**Figure:**
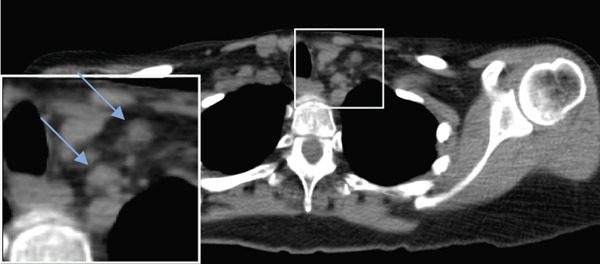
(e)

**Figure:**
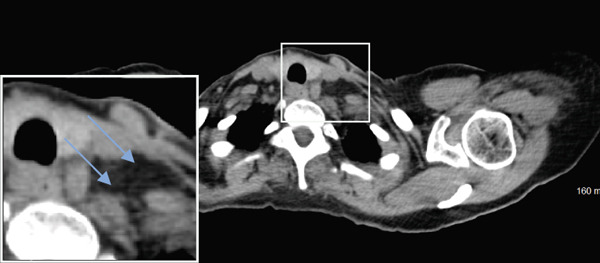
(f)

The patient underwent genomic testing with an expanded panel that revealed the presence of two germline pathogenic mutations. A pathogenic variant, c.8418+5_8418+8del, was detected in the *ATM* gene (https://www.ncbi.nlm.nih.gov/clinvar/variation/VCV000181866.38), and a second pathogenic variant, c.2990_2993del (p.Thr997fs), was detected in the *BRIP1* gene (https://www.ncbi.nlm.nih.gov/clinvar/variation/VCV000234281.65). In addition, two variants of uncertain significance were detected in the *AXIN1* and *BRCA2* genes.

The patient was treated with adjuvant chemotherapy comprising 5FU, leucovorin, and irinotecan (FOLFIRINOX) as previously described [23]. The patient was monitored, and 1 year later, restaging revealed two new supraclavicular lesions measuring 10 and 8.8 mm, respectively, at the longest diameter, which was found to be consistent with metastatic pancreatic carcinoma (Figure 2b,e). Consequently, the patient was retreated with FOLFIRINOX chemotherapy, and the patient achieved a complete remission after 12 cycles (Figure 2c,f). Due to the patient′s known homologous recombinant deficiencies (evidenced by her copathogenic mutations of the *ATM* gene and *BRIP1* genes), 300 mg of olaparib was given orally twice a day, which was used off‐label as maintenance therapy. Duration of response was measured from the start of olaparib treatment. The patient has since remained in complete remission and her most recent restaging PET/CT scan shows an ongoing complete remission at approximately 55 months of therapy with maintenance olaparib (Figure 2c,f). Evaluation of ctDNA for minimal residual disease using the Signatera platform (Signatera, Natera Inc.) has continued to demonstrate the absence of detectable disease in the patient at a molecular level. Biochemical evaluation is notable for normalized tumor markers, including the levels of her CA 19‐9 (Table 1). CA 19‐9 levels above 37 U/mL are generally considered elevated and warrant further testing, and only the first CA‐19‐9 measurement on 2/22/2019 was elevated. Additionally, the patient has remained free of recurrence from her breast carcinoma and her ovarian carcinoma (Figure 1).

**Table 1:** CA 19‐9 measurements.

**Date of test**	**CA-19-9 level (U/mL)**
2/22/2019	40.0
3/16/2019	23.0
6/03/2019	24.4
7/9/2019	27.6
9/25/2019	19.9
10/14/2020	8.5
2/24/2021	14.3
3/17/2021	22.5
10/20/2025	12.3

## 3. Discussion

Metastatic pancreatic carcinoma is associated with a poor prognosis. In the maintenance olaparib for germline *BRCA1* mutated metastatic pancreatic carcinoma study (POLO‐1), olaparib maintenance exhibited a progression‐free survival of 7.4 versus 3.8 months for patients treated with a placebo, with a hazard ratio (HR) of 0.53 [3]. At the time of the initial interim survival analysis, no differences in survival were seen, with a median of 18.9 versus 18.4 months and a HR of 0.91. In the final analysis of the same data published in the *Journal of Clinical Oncology* in 2024 [24], no statistically significant overall survival benefit was seen. However, the HR numerically favored olaparib with a 3‐year survival of 33.9 months for olaparib versus 17.8 months for the placebo group. Survival of patients with pancreatic cancer following relapse after adjuvant FOLFORINOX chemotherapy is unknown, but current estimates for the median survival of patients following relapse with metastatic PDAC after adjuvant therapy are estimated to be around 7 months (95% CI, 6–9 months) [25].

The clinical outcomes for our reported case are notably superior to those currently reported in the literature, based on the median expected overall survival rates. We speculate that this patient, who has remained in an ongoing complete remission on olaparib therapy, has demonstrated a more than expected progression‐free survival due to the combination of two mutations involving the homologous recombination pathway with resultant deficiencies in the pathway, which makes the patient particularly sensitive to platinum‐based chemotherapy. Therefore, retreatment with FOLFIRINOX for this patient resulted in the achievement of a complete remission, and the resultant maintenance with olaparib continued to prevent DNA repair in the neoplastic cells, thereby preventing a recurrence. There is growing evidence suggesting a potential clinical benefit of PARP inhibitors in patients with one of the classical HRD‐associated cancer types, in whom a *BRIP1 mutation* has been identified [26]. Complete remission was recently reported for the first time following olaparib treatment in a patient with BRIP1‐mutated metastatic sarcoma [27].

The current standard of care in ovarian carcinoma for patients with HRD deficiencies is routine maintenance with PARP inhibitors following platinum‐based chemotherapy, which has shown to improve outcomes. However, the current standard of care for PDAC patients with HRD deficiencies continues to be just chemotherapy followed by observation. While there are some ongoing studies evaluating PDAC patients with HRD pathway deficiencies and the effects of PARP inhibitors on patient outcomes, those results are still pending. We believe that this case report confirms the principle of PARP inhibitor sensitivity, specifically olaparib, in patients with PDAC with HRD pathway deficiencies. Therefore, we recommend olaparib be considered as a maintenance therapy for patients with PDAC with HRD pathway deficiencies, pending the results of additional clinical studies to confirm our observation.

## Consent

All the patients allowed personal data processing, and informed consent was obtained from all individual participants included in the study.

## Conflicts of Interest

Ruemu E. Birhiray M.D., Abbvie, Amgen Inc., Array, Biopharma Inc., Astrazeneca, Blue Medicines, Cti, Diachi Sancho, Sobi Pharmaceuticals, E.R. Squibb & Sons L.L.C., Epizyme, Exelixis Inc., Genzyme Corporation, Glaxo Oncology, Incyte Corporation, Ipsen, Johnson & Johnson, Janssen Scientific Affairs Llc, Lilly Oncology, Lilly Usa Llc, Morphosys, Pharmacyclics Llc, Puma Biotechnology, Regeneron, Sanofi, Seagen, Stemline, Takeda, Tg Therapeutics Inc. Maya N. Birhiray M.S., nothing to disclose. Samuel L. Ranger M.S., IQVIA. Vincent L. Flanders M.D., nothing to disclose.

## Author Contributions

All authors equally contributed to data gathering and manuscript preparation.

## Funding

No funding was received for this manuscript.
